# Supplementary material for: Cryo-EM structures from sub-nl volumes using pin-printing and jet vitrification
Source: Nat Commun. 2020 May 22;11:2563. doi: 10.1038/s41467-020-16392-5 (PMC7244535; doi:10.1038/s41467-020-16392-5)
Supplement: Supplementary file 3 — Description of Additional Supplementary Information [file 41467_2020_16392_MOESM3_ESM.pdf]

Description of Additional Supplementary Files

File Name: Supplementary Movie 1
Description: Dewpoint control loop. The effect of changing the feedback offset temperature by 0.5o C increments shown in real time. Condensation and evaporation can be seen when the grid temperature is cooled and, respectively, heated. The 2 minute long movie is shown in real-time. The temperature of the grid, as measured by the copper ring of the autogrid (red) is shown as well the as the calculated dewpoint (blue). Dewpoint calculations are based on the ambient temperature of the deposition chamber along with the relative humidity in the chamber. The temperature of the grid is initially maintained above the calculated dewpoint of 21.2o C at 21.9o C (0- 18s video span) before being decreased to 21.4o C (18-45s), only 0.2o C above the dewpoint temperature. The temperature is further decreased 20.9o C (45-92s), approximately 0.3o C below dewpoint. Condensation on the grid is seen when the temperature of the grid is decreased below the dewpoint based on the droplet formation in the grid squares. Growth of the droplets is seen by Newton rings forming and droplet growth as determined visually by means of a camera behind the grid.

File Name: Supplementary Movie 2
Description: Dewpoint control loop. Same movie as in Supplementary Movie 1, but shown at 5x speed.

File Name: Supplementary Movie 3
Description: Video recorded by the grid inspection camera showing the sample deposition in real time. The presence of a suitable thin layer as well as the occurrence of sample evaporation or condensation can be assessed.

File Name: Supplementary Movie 4
Description: Slow motion video recorded by a high speed camera showing vitrification of an autogrid with two jets of liquid ethane. The jets last for 50 ms.

File Name: Supplementary Movie 5
Description: Animation showing the working principles of the VitroJet.
